# The Pulseq-CEST Library: definition of preparations and simulations, example data, and example evaluations

**DOI:** 10.1007/s10334-025-01242-6

**Published:** 2025-03-27

**Authors:** Alexander Liebeskind, Jan Rüdiger Schüre, Moritz Simon Fabian, Simon Weinmüller, Patrick Schünke, Vladimir Golkov, Daniel Cremers, Moritz Zaiss

**Affiliations:** 1 Computer Vision Group, Technical University of Munich (TUM), Boltzmannstraße 3, 85748 Garching bei München, Germany; 2 Munich Center for Machine Learning (MCML), Oettingenstraße 67, 80538 Munich, Germany; 3 Institute of Neuroradiology, University Hospital Erlangen, Erlangen Schwabachanlage 6, 91054 Erlangen, Germany; 4 Physikalisch-Technische Bundesanstalt (PTB), Braunschweig and Berlin, Bundesallee 100, 38116 Brunswick, Germany; 5 Department of Artificial Intelligence in Biomedical Engineering (AIBE), FAU Erlangen, Erlangen Werner-von-Siemens Str. 61, 91052 Erlangen, Germany

**Keywords:** Magnetic resonance imaging, Computer simulation, Image processing, Computer assisted, Phantoms, Imaging, Software

## Abstract

**Objectives:**

Despite prevalent use of chemical exchange saturation transfer (CEST) MRI, standardization remains elusive. Imaging depends heavily on parameters dictating radiofrequency (RF) events, gradients, and apparent diffusion coefficient (ADC). We present the Pulseq-CEST Library, a repository of CEST preparation and simulation definitions, including example data and evaluations, that provides a common basis for reproducible research, rapid prototyping, and in silico deep learning training data generation.

**Materials and methods:**

A Pulseq-CEST experiment requires (i) a CEST preparation sequence, (ii) a Bloch–McConnell parameter set, (iii) a Bloch–McConnell simulation, and (iv) an evaluation script. Pulseq-CEST utilizes the Bloch–McConnell equations to model in vitro and in vivo conditions. Using this model, a candidate sequence or environment can be held constant while varying other inputs, enabling robust testing.

**Results:**

Data were compared for amide proton transfer weighted (APTw) and water shift and B1 (WASABI) protocols using a five-tube phantom and simulated environments. Real and simulated data matched anticipated spectral shapes and local peak characteristics. The Pulseq-CEST Library supports similar experiments with common sequences and environments to assess new protocols and sample data.

**Discussion:**

The Pulseq-CEST Library provides a flexible mechanism for standardizing and prototyping CEST sequences, facilitating collaborative development. With the capability for expansion, including open-source incorporation of new sequences and environments, the library accelerates the invention and spread of novel CEST and other saturation transfer approaches, such as relayed NOEs (rNOEs) and semisolid magnetization transfer contrast (MTC) methods.

**Supplementary Information:**

The online version contains supplementary material available at 10.1007/s10334-025-01242-6.

## Introduction

Magnetic resonance imaging (MRI) has well-established applications spanning a wide array of practice areas. Chemical exchange saturation transfer (CEST) MRI describes an advanced MRI technique that relies on detecting proton exchange between water and solute molecules in biological tissues. This allows CEST to yield imaging that highlights targeted molecular components and metabolites, granting visibility into tissue properties that can help elucidate brain tumors and other abnormalities.

Given the importance of optimizing MRI approaches, a significant body of research has been dedicated to understanding pulse sequences, Bloch–McConnell simulations, and evaluation methods. Since it can often be more straightforward to work with pulse sequences prior to deployment within a physical MR machine, open-source frameworks have emerged that allow for the development of pulse sequence parameters in silico. Pulseq, for example, supports pulse sequence programming in MATLAB and Python, generating text files that work in simulation but also in MR scanners [1]. While image reconstruction functions are commonly vendor-specific and proprietary, it is possible to use tailored Pulseq interpreters with Pulseq pulse sequences to provide compatibility with established MRI readouts [e.g., fast low angle shot (FLASH), gradient-recalled echo (GRE), echo planar imaging (EPI)] [2]. In the context of CEST, Pulseq is, therefore, ideal because it delivers portability, transparency, and therefore reproducibility: CEST preparation periods are accessible and interpretable, easy to work with in prevalent programming languages, directly transferable to MATLAB or Python simulations, and readily functional in real scanners.

As the usage of different parameter sets for CEST imaging—including specifications such as irradiation strength (B1), total saturation time ($t_{sat}$), saturation duty cycle (DC), radiofrequency (RF), pulse shape (magnitude, phase), pulse duration ($t_p$), pulse delay ($t_d$), and phase cycling ($\phi _i$)—has not been standardized so far, an important step for quantitative and comparable CEST imaging is standardization to prevent the emergence of different signals [3, 4]. To facilitate this task, Pulseq-CEST [2] was launched to create a consensus on preparation methods and encourage sharing of CEST parameters, though efforts to standardize CEST methodology remain a work in progress [5, 6]. As a result of the Pulseq-CEST open standard, researchers have been able to share pre-saturation schemes in a recognized format in various studies [3, 7, 8]. Beyond enhancing reproducibility, Pulseq-CEST data have also been used in deep learning as ground truth [9]. This application is increasingly important given the growing usage of deep learning in MR sequencing and the essential nature of a baseline consensus as a means of evaluating model performance.

Achieving standardization with Pulseq-CEST requires accounting for multiple components. Starting from the pulse sequences, it is essential to set up established protocols including the specified CEST sequences (e.g., amide proton transfer weighted (APTw) [10]), which can optionally be followed by a water shift and B1 (WASABI) sequence to perform B0 and B1 correction in post-processing [11]. These protocols must then be applied to consistent environments, including real and simulated data. In other to maintain cross-study comparability and reproducibility, the evaluation scripts used must also involve the same steps.

The Pulseq-CEST Library, building upon the introduction of the initial Pulse-CEST standard to drive towards a more robust understanding of CEST, provides sequences, simulation environments, phantoms, and evaluation methods to streamline working with CEST across protocols. These examples provide a common basis for working with popular pulse sequences and simulation environments, laying the foundation for more universal and reproducible MRI research across CEST and other saturation transfer approaches including relayed NOEs (rNOEs) and semisolid magnetization transfer contrast (MTC) methods [12]. This work focuses on providing demonstrations with the CEST effect.

Using the Pulseq-CEST Library, one candidate sequence or environment can be thoroughly tested while changing the other input parameters. This allows for the various components of a Pulseq-CEST simulation to be standardized, or for new sequences and environments to be developed and shared. For example, if an L-arginine environment is the object for CEST investigations, the simulation environment can be held constant while varying between different sequence parameters. Other sequences can also be deployed to further investigate the simulation environment in the context of protocols for glutamate [13], pH-weighted [14, 15], WASABI [16], or dynamic glucose enhanced (DGE) [17] at different field strengths such as 3T and 7T. This allows for comparison of entire protocols and discrete evaluation of individual changes—a user could determine how changing the number of pulses or flip angle directly changes the resulting spectra and parameter maps for instance. When designing new contrasts, this ability is critical, as novel pulse sequences can be designed and tested in silico without necessitating a real scanner. Due to the format of Pulseq files, a resulting sequence can then be used in scanners across vendors, allowing for confirmation of simulated results in phantoms and in vivo.

**Fig. 1:**
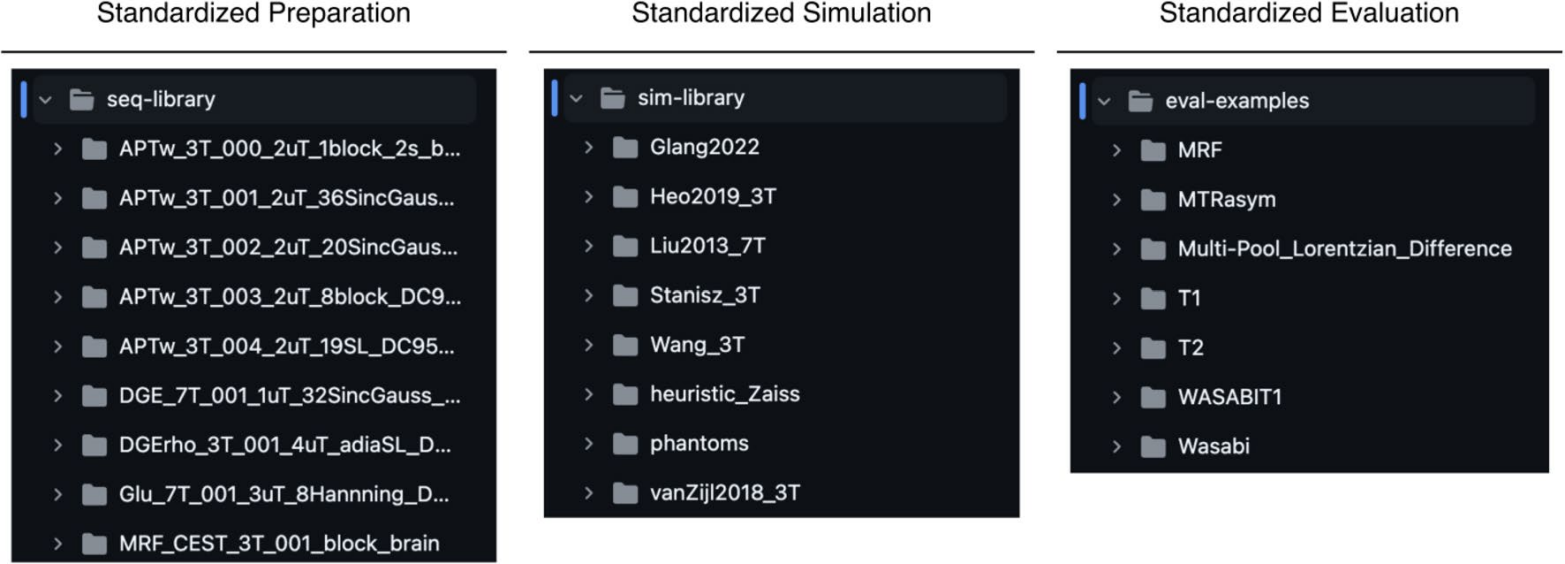
Illustration of the Pulseq-CEST pipeline, which is based on Pulseq. Standardized preparations, simulations, and evaluations provide the basis for reproducible experiments that can be replicated across simulated environments and vendor-specific hardware using tailored interpreters [18]

A major benefit of this approach is therefore cross-vendor compatibility. Beyond the pragmatic considerations mentioned above, developing and experimenting with pulse sequences prior to interpretation provides portability across manufacturers, allowing for a hardware independent approach. The experimental data can then be used to further iterate upon Pulseq sequence files. In practice, the Pulseq-CEST library can be divided into three sections as demonstrated in Fig. 1. The full library is accessible to the public through the repository at https://github.com/kherz/pulseq-cest-library. while the code demonstrations used here are located https://github.com/kherz/pulseq-cest-library/releases/tag/MAGMA_2025_PAPER. The associated phantom data can be found at https://zenodo.org/records/15053086.

The library can be utilized to simulate distinct chemical exchange mechanisms given the differing use cases for MRI protocols. APT-weighted imaging, for instance, is commonly used to identify tumors exhibiting unusual pH levels [15], elevated protein contents [19], or increased cellularity in high-grade gliomas. In this type of imaging, a series of RF pulses is applied in a symmetrical manner offset from water resonance with additional saturation parameters [3], provided in the seq-files of the seq-library. These parameters can be adjusted for each specific task or for standardization. In addition to the APT-weighted protocols or other examples like glucose-weighted CEST (glucoCEST), B0 and B1 correction can be performed according to the WASABI sequence [11, 20]. Furthermore, the sim-library offers various environments in which the respective seq-files can be simulated, while the eval examples provide a simple evaluation of the respective sequences.

In short, the Pulseq-CEST Library makes it easier not only to measure, but also to simulate and experiment with imaging techniques that can be used to target specialized physiology. In the following work, we concentrate on the evaluation of CEST data.

## Materials and methods

### Theory

To fully understand the Pulseq-CEST Library, it is necessary to investigate the principles underlying CEST theory. In CEST MRI, frequency-selectively RF irradiation is employed to saturate the protons of a functional group featured in a target molecule pool of interest. By measuring the corresponding changes in the water pool magnetization due to chemical exchange with the solute proton pool, CEST obtains a signal that depends on the concentration of the solute and affected by other factors—such as pH and buffer concentration in particular—that impact exchange rate. This signal may be indicative of altered metabolism and tissue properties. Prior to the RF preparation, it is also necessary to allow magnetic nuclei in the sample to return to their equilibrium state between each offset by inserting a relaxation time, which helps to ensure reliability and consistency of results.

A CEST sequence, thus, involves three aspects: (1) a relaxation delay, (2) an RF preparation period for saturation of solute molecules at various frequencies, and (3) an acquisition observing changes in the water pool magnetization. To prevent unwanted signals and artifacts (e.g., blood flow artifacts, fat signal) from interfering with the CEST signal, a crusher gradient is additionally applied between the RF preparation and acquisition, leaving only the Z-component of the water pool magnetization. This allows for the computation of a Z-spectrum, normalizing signal at each frequency offset ($\Delta \omega$) in comparison to the unsaturated scan $S_0$:

1 $$\begin{aligned} Z(\Delta \omega ) =S_\textrm{sat} (\Delta \omega ) / S_0. \end{aligned}$$

CEST image collection can be conducted through post-processing using this Z-spectrum, which emerges from repeating step (1)–(3) at different off-resonant frequencies. Because we retain readout parameters constant throughout the CEST experiment, the Z-spectrum is dictated by RF irradiation parameters such as amplitude, offset, and duration, as well as B0 and B1 field strength. Especially for the last two cases, field homogeneity is crucial in CEST imaging since changes in these fields can cause errors, either in the frequency information of an offset and/or the correct saturation amplitude.

### Application

#### Simulation

The Bloch—McConnell simulation of Pulseq-CEST is based on a piecewise constant approximation of CEST pulses using continuous wave saturation at the same B1 level [21, 22]. It uses the Padé algorithm [23] to compute the occurring matrix exponential stepwise, so it can also be used as a simulation algorithm for trains of shaped pulses. The Pulseq-CEST simulation has a dynamic matrix size, which means that an arbitrary number of exchanging pools, including semi-solid magnetization transfer (MT), can be modeled. The simulation was recently validated via an international comparison [24] of 11 different Bloch–McConnell solvers.

#### Pulse sequences

In this work we used an APTw 3T Sinc Gauss pulse sequence ($t_p$ = 50 ms, $t_d$ = 5 ms, number of pulses = 36, DC = 91%, $t_\textrm{sat}$ = 1.975 s, B1 = 2 $\upmu$T) protocol from the Pulseq-CEST Library and further used a WASABI sequence ($t_p$ = 5 ms, number of pulses = 1, $t_\textrm{sat}$ = 5 ms, B1 = 3.7 $\mu$T) to correct for B0 and B1 inhomogeneities [11]. With WASABI, we find the B0 information in the water peak position and the B1 information in the properties of the Rabi oscillations. The pulse sequences were used to determine resulting spectra and parametric maps across real phantom and simulated data.

#### Phantom and simulated data

The phantom tubes were designed to mimic healthy tissue (1$\times$), tumor tissue (2$\times$), and liquid cysts (2$\times$). Therefore, five 50 mL Falcon tubes were filled with 3.9 $\upmu$L Gadovist at a concentration of 1 mM to reach a T1 of approximately 1500 ms. Different amounts of agarose from 0–2% were added to provide the specific tissue types. Since the tumor and reference tissue phantoms contain semi-solid compartments, 2% agarose was added to the heated model solution. For the CEST effect, different concentrations of L-arginine (20–70 mM) were added [25]. To ensure a chemical exchange rate of about 350 Hz [25, 26], a pH value of approximately 4.0 (exchange rate estimated from supporting Fig. S1) was titrated using hydrochloric acid (HCl) in a 0.9% sodium chloride (NaCl) solution. The phantom parameters are given in Table 1.

**Table 1:** Detailed metrics on phantom tube composition

Tube	Type	Gadovist [1 mM]	Agarose	L-Arginine
Tube 1	Cyst-like	3.9 $\upmu$L	0%	20 mM
Tube 2	Cyst-like	3.9 $\upmu$L	0%	27 mM
Tube 3	Tissue-like	3.9 $\upmu$L	2%	40 mM
Tube 4	Tumor-like	3.9 $\upmu$L	2%	60 mM
Tube 5	Tumor-like	3.9 $\upmu$L	2%	70 mM

Description of the characteristics of five 50 mL Falcon tubes included in the phantom, producing different chemical exchange properties for CEST experimentation [25]

#### Process

The experiments were performed in Python. Using the described Pulse-CEST Library pulse sequences, environments, and evaluations, we demonstrate experimentation with a single pulse sequence (i.e., APT-weighted) and then with a single environment (i.e., L-arginine). This allowed for comparison of the effects of swapping the pulse sequence or data independently, while adapting the relevant evaluation scripts. For the evaluation, we used a region-of-interest (ROI)-based approach.

## Results

### Overview and demonstration

The process requires several inputs: a sequence file, input data (either real or simulated), and specifications that dictate how the evaluation should occur. For instance, the evaluation can be tuned to select for a ROI; in this study, this method is used to select one of the phantom tubes.

**Fig. 2:**
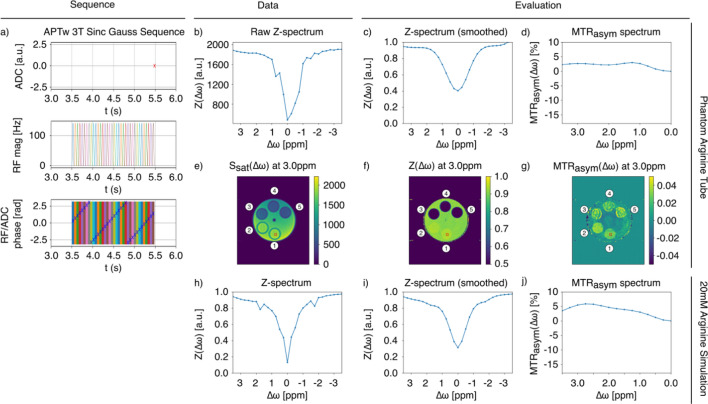
Comparison of real and simulated arginine CEST experiments. Pulseq-CEST pipeline demonstration using an APTw 3T Sinc Gauss pulse sequence to compare a selected tube within a real five-tube phantom (top, **b**–**g**) with simulated L-arginine data (bottom, **h**–**j**) (3T, 20 mM, pH 4, T1 = 1500 ms, T2 = 1000 ms). The illustration shows **a** the complex sequence diagram summarizing the apparent diffusion coefficient (ADC) event, RF irradiation after 3.5 s Trec, and corresponding phase information for each RF pulse, **b** the raw Z-spectrum prior to evaluation, **c**, **d** the Z-spectrum and MTRasym plot for the selected area of the L-arginine tube marked in red, **e**–**g** the parametric plots generated for raw (i.e., the saturation plot) and evaluated data, **h** the Z-spectrum for the 20 mM L-arginine simulation, and **i**, **j** the Z-spectrum and MTRasym plot for the simulated L-arginine environment with smoothing interpolation. Tube numbers are also labeled in the phantom as dictated by Table 1. The simulation parameters for h)—j) can be found here: https://github.com/kherz/pulseq-cest-library/blob/master/sim-library/phantoms/l-arginin/L-arginin_3T_20mM_pH4_T1_1500ms_T2_1000ms_bmsim.yaml. Each subfigure can be recreated with the corresponding letter using Figure2.py found at https://github.com/kherz/pulseq-cest-library/releases/tag/MAGMA_2025_PAPER

This pipeline more specifically works as shown in Fig. 2, which demonstrates the example of the described APTw 3T Sinc Gauss pulse sequence with our real five-tube phantom and a simulated L-arginine environment. Comparing real and evaluated data for the 20 mM tube demonstrate similar Z-spectra shapes across the real and simulated data, as well as magnetization transfer ratio asymmetry (MTRasym) plot peaks at approximately +3 ppm as associated with L-arginine. This indicates alignment between results.

**Fig. 3:**
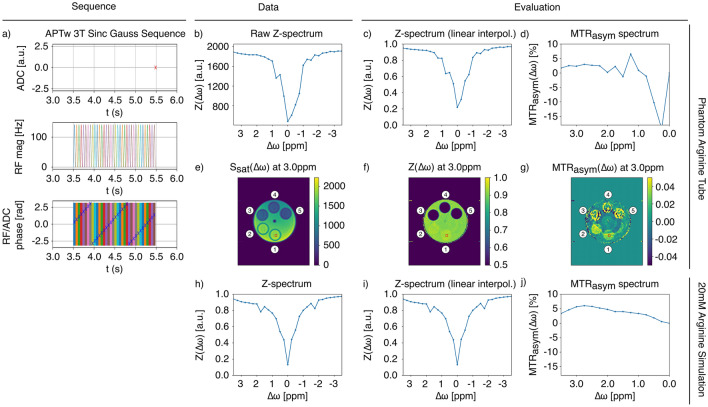
Demonstrating the affect of changing only the evaluation script. Pulseq-CEST pipeline demonstration using an APTw 3T Sinc Gauss pulse sequence and five-tube phantom with linear interpolation rather than smoothing spline interpolation. The illustration shows **a** the complex sequence diagram summarizing the ADC, RF irradiation after 3.5 s Trec, and corresponding phase information for each RF pulse, **b** the raw Z-spectrum prior to evaluation, **c**, **d** the Z-spectrum and MTRasym plot for the selected area of the L-arginine tube marked in red, and **e**–**g** the parametric plots generated for raw (i.e., the saturation plot) and evaluated data, **h** the Z-spectrum for the 20 mM L-arginine simulation, and **i**, **j** the Z-spectrum and MTRasym plot for the simulated L-arginine environment with linear interpolation. Tube numbers are also labeled in the phantom as dictated by Table 1. The simulation parameters for **h**–**j** can be found here: https://github.com/kherz/pulseq-cest-library/blob/master/sim-library/phantoms/l-arginin/L-arginin_3T_20mM_pH4_T1_1500ms_T2_1000ms_bmsim.yaml. Each subfigure can be recreated with the corresponding letter using Figure3.py found at https://github.com/kherz/pulseq-cest-library/releases/tag/MAGMA_2025_PAPER

However, we also observe an asymmetry at +0.75 ppm in the true L-arginine tube that is not reproduced in the simulation. As shown in Fig. S2, this artifact in both Figs. 2 and 3 is likely a B0 artifact, as sidebands and B0 shifts can occur when scanning with liquid phantoms. By observing the measured raw Z-spectrum (b) we detect a sideband which potentially influences the MTRasym plot (d). When simulating the phantom data with the same sequence (h), we can also detect these sidebands here. In this manner, analysis of the phantom and simulated spectra allows for reproducible identification of characteristics. The parametric plots (e, f, g) provide an additional means of validating results by illustrating the phantom and the regions of high and low signal intensity at a specific frequency offset.

The evaluation files themselves are considered to be modular and modifiable to a certain extent, supporting changes like combining WASABI B0 correction with MTR asymmetry evaluation. The usage of different interpolation methods also influences the Z-spectra and corresponding MTRasym (c, d), even if the same sequence parameters and data were used as can be observed between Figs. 2 and 3. Here, we observe that smoothing spline interpolation leads to a more regular Z-spectrum while linear interpolation more closely preserves local deviations. While smoothing spline interpolation is often preferred due to the flexibility conferred by fitting with piecewise polynomials, this can obscure small effects in the simulated spectrum. In assuming a linear relationship between points, linear interpolation approach maintains the integrity of the simulated example, though the MTRasym-spectrum appears much less smooth.

### Experimenting with environments

**Fig. 4:**
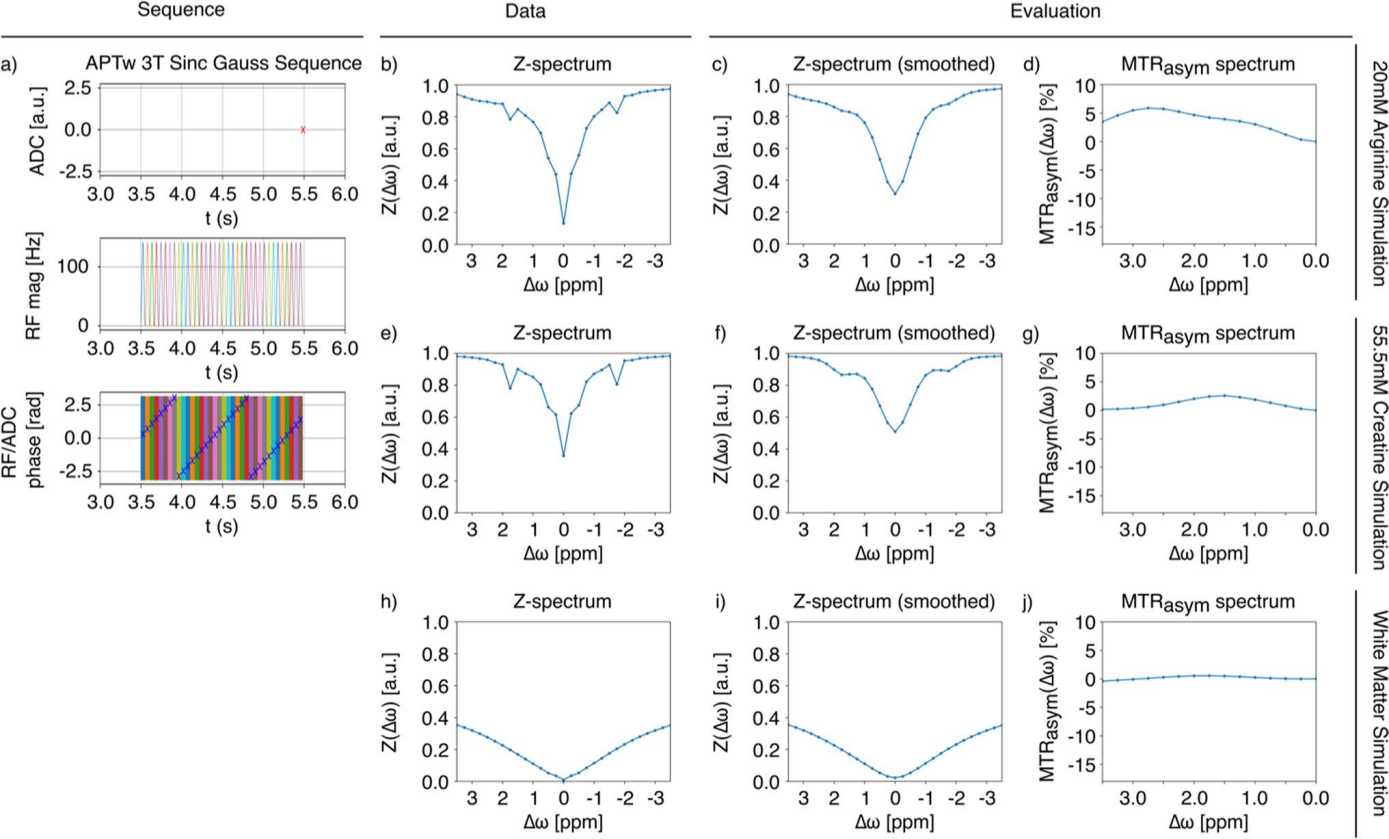
Comparison of different simulation environments given by bmsim.yaml files. Comparison of APTw 3T Sinc Gauss sequence and effects on various simulated environments, including L-arginine (3 T, 20 mM, pH 4, T1 = 1500 ms, T2 = 1000 ms), creatine (3 T, 55.5 mM, pH 6.4, T1 = 3000 ms, T2 = 2000 ms), and white matter (4 CEST pools: amide = 0.65 mM, guanidine = 1.4 mM, amine = 0.18 mM, OH = 0.18 mM, 1 NOE pool: NOE = 4.5 mM). As in Figs. 2 and 3, the illustration shows **a** the complex sequence diagram summarizing the ADC, RF irradiation after 3.5 s Trec, and corresponding phase information for each RF pulse, while here Fig. **b**–**j** show resulting Z-spectra and MTRasym spectra for comparison across simulations. All yaml files and further simulation files used in this paper are provided on Github: https://github.com/kherz/pulseq-cest-library/tree/master. More specifically, the following sources can be used to view simulation parameters for the environments here: (1) https://github.com/kherz/pulseq-cest-library/blob/master/sim-library/phantoms/l-arginin/L-arginin_3T_20mM_pH4_T1_1500ms_T2_1000ms_bmsim.yaml, (2) creatine, (3) https://github.com/kherz/pulseq-cest-library/blob/master/sim-library/WM_3T_001_bmsim.yaml. Each subfigure can be recreated with the corresponding letter using Figure4.py found at https://github.com/kherz/pulseq-cest-library/releases/tag/MAGMA_2025_PAPER

When designing a pulse sequence definition, it can be useful to evaluate simulated data with varying chemical properties. In Fig. 4, the APTw 3T Sinc Gauss pulse sequence is simulated across three environments from the sim-library: L-arginine (3 T, 20 mM, pH 4, T1 = 1500 ms, T2 = 1000 ms), creatine (3 T, 55.5 mM, pH 6.4, T1 = 3000 ms, T2 = 2000 ms), and white matter (4 CEST pools: amide = 0.65 mM, guanidine = 1.4 mM, amine = 0.18 mM, OH = 0.18 mM, 1 NOE pool: NOE = 4.5 mM, with varying T1 and T2 for each pool). The three simulations reveal MTRasym spectra with local peaks corresponding to each chemical compound, at about +3ppm, +1.4 ppm, and +1.9 ppm for arginine, creatine, and white matter, respectively. When comparing to real data, this consideration can provide additional information beyond the frequency offset at which peaks appear.

The simulated data here are defined within the yaml file. In this yaml file, the water pool and the relative CEST pool, the pool size fractions T1 and T2 values as well as the respective exchange rates and the chemical shift towards water can be determined depending on the molecule used. In addition to the settings for the pool model, it is possible to make further adjustments to the strength of the B0 field and to the B0/B1 inhomogeneities.

### Experimenting with pulse sequences

Much like maintaining an individual pulse sequence and varying the environment, the Pulseq-CEST Library also allows for experimentation with various pulse sequences within the same environments, enabling the user to determine the resulting spectra caused by changing protocols. The Pulseq-CEST Library currently supports diverse protocols including but not limited to DGE, GluCEST, MRF, and pH-weighted scanning by providing pulse sequence definitions.

**Fig. 5:**
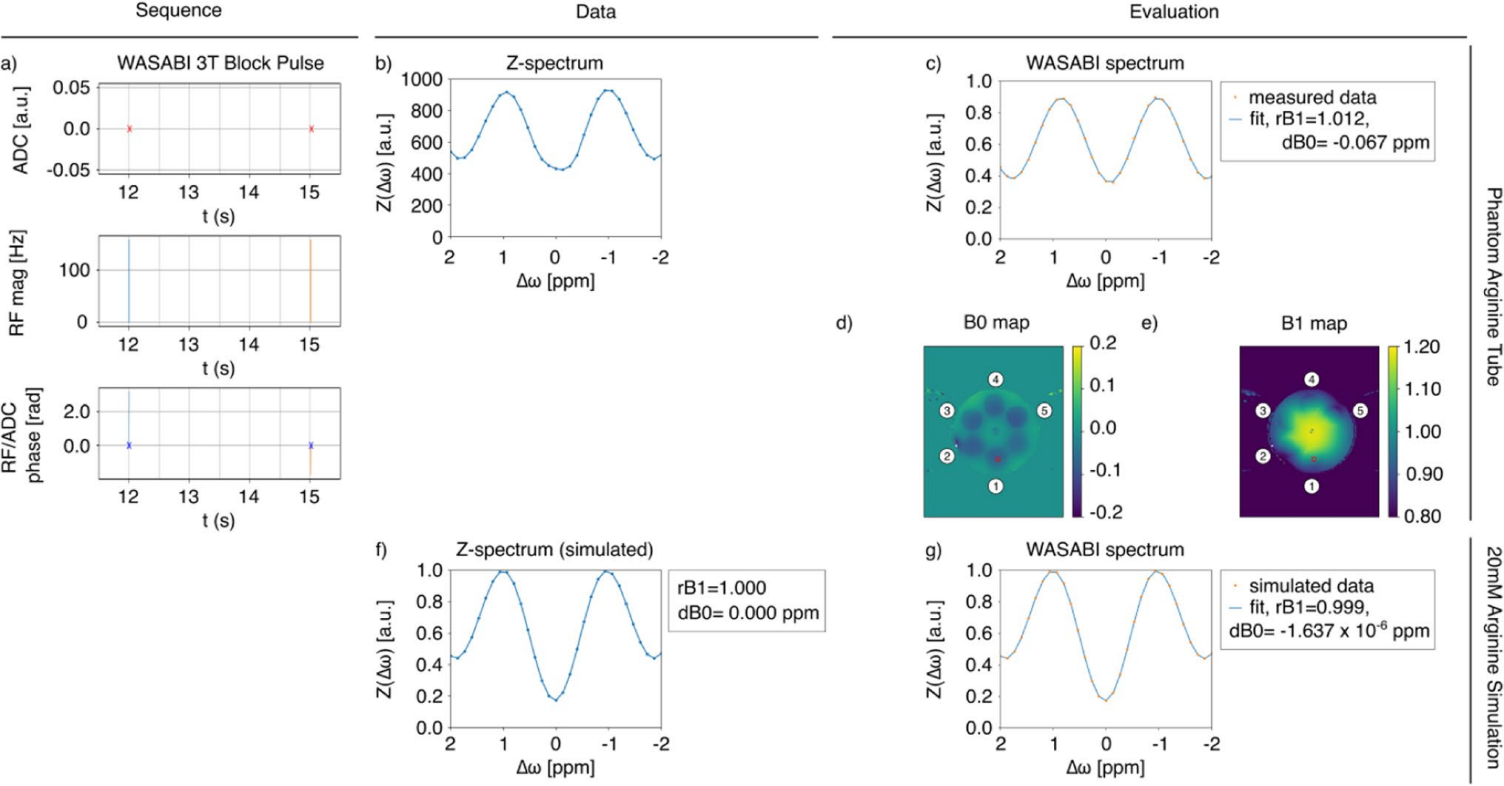
Changing the sequence file to a WASABI pulse sequence. All other parameters remain fixed. WASABI pulse sequence with both a five-tube phantom and simulated L-arginine data (3 T, 20 mM, pH4, T1 = 1500 ms, T2 = 1000 ms). The illustration shows **a** the complex sequence diagram summarizing the ADC, RF irradiation between 12–15 s Trec, and corresponding phase information for each RF pulse. Parts **b**–**j** show the resulting spectra and parametric maps for the WASABI data, with the selected area of the L-arginine tube marked in red. Tube numbers are also labeled in the phantom as dictated by Table 1. Each subfigure can be recreated with the corresponding letter using Figure5.py found at https://github.com/kherz/pulseq-cest-library/releases/tag/MAGMA_2025_PAPER

In addition to the APTw 3T Sinc Gauss, the following example demonstrates the possibility of testing other MR sequences such as WASABI, which is, in general, used for the correction of field inhomogeneities for CEST data. This is illustrated in Fig. 5, which compares the Pulseq-CEST output for a simulated WASABI sequence for the corresponding voxel positions of the L-arginine simulation as shown in Fig. 2 against the measured data. This results in the creation of B0 and B1 maps, which can provide a means of better understanding B0 fluctuations and conducting shifting or shimming.

In Fig. 5, the spectra produced by the phantom L-arginine tube closely resemble simulated results, reflecting the characteristic shape of the WASABI fit. As shown, the simulated environments furnished by the Pulseq-CEST Library can be used across protocols to standardize pulse sequence definitions and phantom creation. Beyond providing a benchmark for sequence experimentation, this provides a valuable tool for comprehensively understanding the analytical properties of targeted environments, as is observed for the five-tube phantom.

## Discussion

### Prior work

Despite efforts to accurately describe the CEST methods deployed in existing research, there remains a need to reproduce results from prior experiments and further compare the reproducibility of CEST approaches [4, 27, 28]. As visualized in Figs. 2 and 5, results sometimes do not match even across simulation and real-world data, as actual data present spectra irregularities originating from sidebands or field inhomogeneities. In general, this problem could be minimized by selecting a larger ROI and averaging the deviations, but this may also miss artifacts such as sidebands. Modulating between methods of interpolation can further help to better understand these spectra irregulaties, as spline interpolation smooths out artifacts, but linear interpolation retains accuracy. It is also possible to anticipate these effects and produce standardized sequences and environments to analyze this issue. In this work, we witnessed simulated sidebands in creatine and L-arginine simulations, demonstrating the simulation of potential artifacts. As new contributors add to the open-source repository and novel artifacts are reproduced, the existing components of the Pulseq-CEST library can be modified to account for fresh discoveries, further solidifying validation capabilities.

**Fig. 6:**
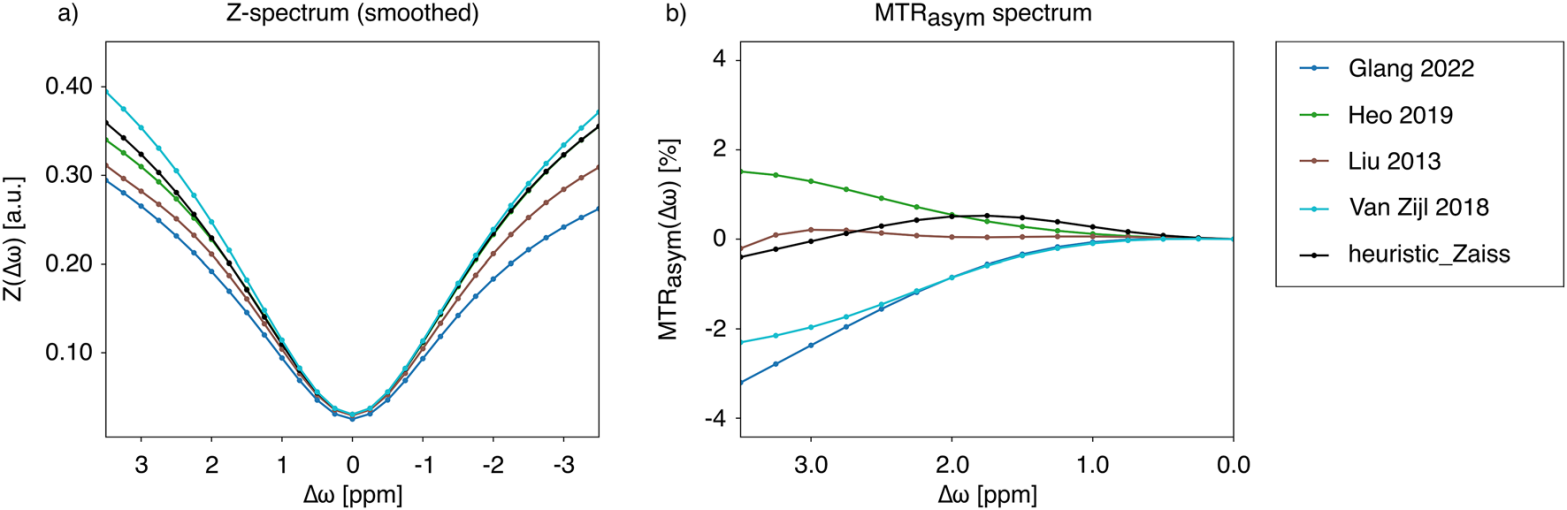
Divergence between simulated white matter spectra using experimental parameters from different studies. Output spectra comparisons for amide proton transfer weighted (APTw) imaging, showing divergence across past studies [29–32] in white matter environment specifications. **a** Illustrates respective Z-spectra, while **b** compares the MTRasym plots, showing discrepancies across both spectrum types. Each subfigure can be recreated with the corresponding letter using Figure6.py found at https://github.com/kherz/pulseq-cest-library/releases/tag/MAGMA_2025_PAPER

The issue of variation without standards is also exacerbated by varying definitions across studies. As illustrated by the Z-spectra and MTRasym spectra in Fig. 6, comparisons between reproduced sequences can show significant deviation between experiments, which makes it difficult to reach consensus on simulation parameters. This can stem from any part of the pipeline, as spectra are directly influenced by the parameters of the sequence, data, and evaluation steps. In this context, the Pulseq-CEST Library can serve as a standardization tool that can be adapted and expanded to accommodate modifications to protocols. Beyond highlighting discrepancies and allowing for correction, the pipeline also has the critical functionality of isolating where deviations are arising from, and whether the parameters of the sequence, data, or evaluation need to be adjusted to reach alignment between results.

To stay up to date, the Pulseq-CEST Library is built to accommodate additional sequences, environments, and phantoms, forming the basis for future expansion. As shown with the APTw and WASABI sequences, maintaining openness to new protocols in this manner can help to understand different properties of a given phantom, as parametric maps (including B0 and B1 maps) can be derived.

The idea of using the Pulseq-CEST standard has been applied in several studies already, including a comprehensive CEST study [25] and a recently published paper about fluid suppression [25] where the Pulseq-CEST Library allowed researchers to compare the impact of a correction method in simulation and phantom data. The Pulseq-CEST Library also made it possible to show that real measured sidebands also appear in simulated data [4], opening the possibility for mitigating undesired effects. This example demonstrates the potential that standardization provides to explore MR-effects and rapidly try out new solutions to specific problems [4].

As a result of the lack of a uniform pulse sequence and simulation standard, other initiatives have similarly aimed to facilitate collaborative development and reproduction of CEST pulse sequences and environments. The Bloch–McConnell simulation (BMsim) challenge [24], for example, invited research teams to engage with simulating APT weighted and WASABI use cases, aggregating and evaluating all results online for comparison. Even with a set of general settings and assumptions, live time evaluation files indicated deviation between pulse parameters used by research groups and proved how input differences can impact the resulting simulations.

### Conclusion

In the course of this work, we presented the possibility of using a Pulseq standard in CEST experiments, namely the Pulseq-CEST framework. As the standardization of pre-saturation scheme, simulation, measurement and evaluation improves transparency and reproducibility of CEST acquisitions, further studies involving multiple sites can be deemed possible.

Beyond optimizing the pulse sequences, environments, and phantoms used to generate simulated signals, the Pulseq-CEST Library strives for compatibility with open-source image processing resources that can help to improve Pulseq-CEST from an imaging perspective. The Medical Imaging Interaction Toolkit (MITK) [33], for example, offers a broad suite of image processing capabilities related to viewing, analyzing, and segmenting images, which can be combined with simulation tools to inform sequence development and refine standards. Alongside breakthroughs in CEST methodology and novel applications in deep learning and related fields, the Pulseq-CEST Library be used in conjunction with other imaging resources to encourage more concrete standards.

## Supplementary Information

Below is the link to the electronic supplementary material.

Supplementary file 1 (pdf 162 KB)

## Data Availability

The Pulseq-CEST Library, including the sequence definitions, environments, and evaluation scripts used in this research study, can be openly accessed at https://github.com/kherz/pulseq-cest-library. The code demonstrations used in this paper can additionally be found at https://github.com/kherz/pulseq-cest-library/releases/tag/MAGMA_2025_PAPER. The associated phantom data can be found at https://zenodo.org/records/15053086. Any subsequent updates will also be added to this repository to ensure ongoing consistency.
