# Supplementary material for: The Pulseq-CEST Library: definition of preparations and simulations, example data, and example evaluations
Source: MAGMA. 2025 Mar 27;38(3):413–22. doi: 10.1007/s10334-025-01242-6 (PMC12255581; doi:10.1007/s10334-025-01242-6)
Supplement: Supplementary file 1 — (pdf 162 KB) [file 10334_2025_1242_MOESM1_ESM.pdf]

Appendix A Supplementary Materials

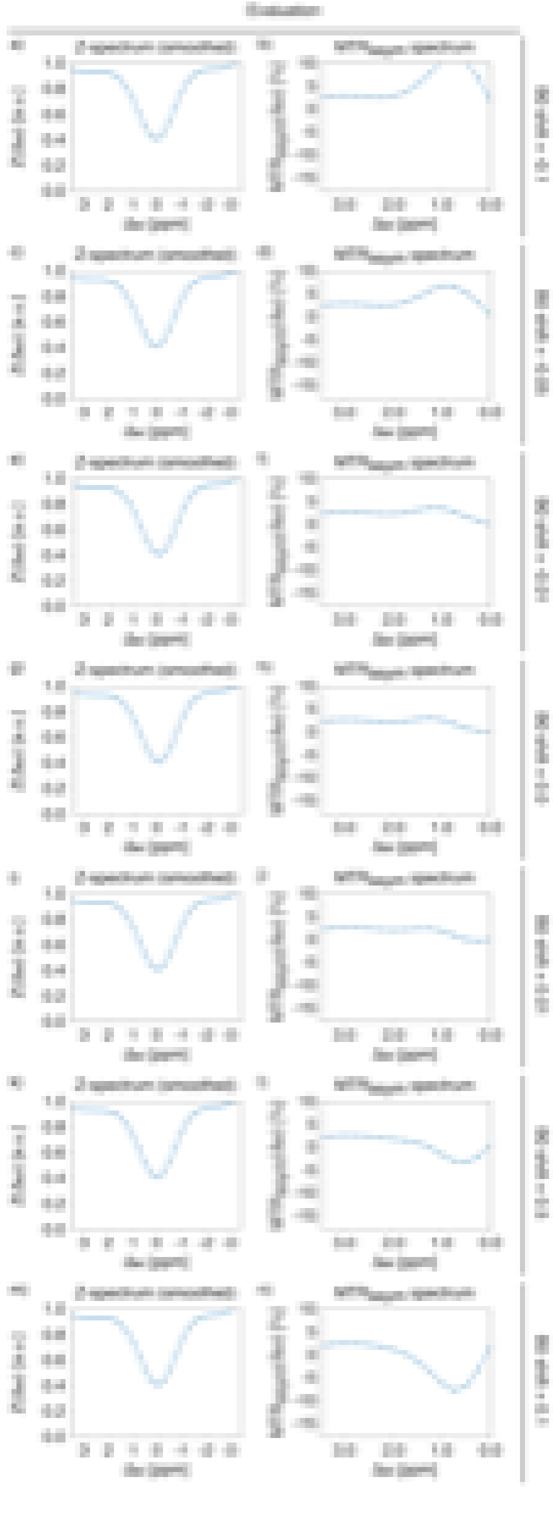

Fig. S1 Illustration of B0 artifacts demonstrated through successive B0 shifts in an L-arginine phantom. The environment is L-arginine (3T, 20mM, pH4, T1 = 1500ms, T2 = 1000ms). Plots demonstrate Z-spectra and MTRasym spectra across B0 shifts of 0.1 (a,b), 0.05 (c,d), 0.01 (e,f), 0.0 (g,h), -0.01 (i,j), -0.5 (k,l), and -0.1 (m,n). As a result, the B0 artifact induced peak at ∼+0.7ppm changes in both magnitude and direction, but the L-arginine peak at ∼+3ppm does not change.

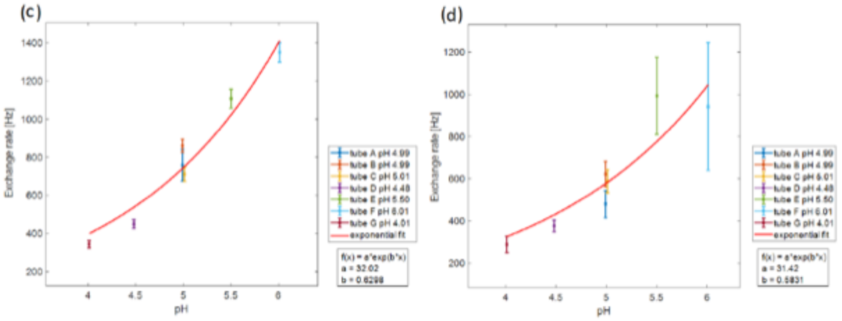

Fig. S2 Exchange rate estimation over variations in pH level. Regression plots indicate mean values inside a region-of-interest (ROI) for different concentrations of L-Arginine [A=25mM,B=100mM,C-G =50mM] acquired at different pH levels [A=4.99, B=4.99,C=5.01,D=4.48,E=5.50,F=6.01,G=4.01]. L-Arginine was dissolved in a pH 4.0 buffer at the specified concentrations and adapted with NaOH to reach pH levels of 4.0 , 4.5, 4.0, 5.5, 6.0. 6g of salt were added in the water for better shimming. The temperature of the solution was measured to be 25°C. Data were acquired with a standard MRF-protocol with a) an EPI and b) with a GRE readout [26].
